# Organoleptic properties and perception of maize, African yam bean, and defatted coconut flour‐based breakfast cereals served in conventional forms

**DOI:** 10.1002/fsn3.336

**Published:** 2016-01-20

**Authors:** Grace Ojali Usman, Gabriel Ifeanyi Okafor

**Affiliations:** ^1^ Department of Food Science and TechnologyUniversity of NigeriaNsukkaEnugu StateNigeria

**Keywords:** Attribute perception, consumption with milk or water, malt extract, sensory profile

## Abstract

Breakfast cereals were produced by roasting (*t* = 280°C) – a dry heat treatment process to gelatinize and semidextrinize the starch – in order to generate dry ready‐to‐eat products from blends of African yam bean (AYB), maize (M), and defatted coconut (DC) flour. Six samples were generated by mixing AYB and maize composite flour with graded levels of DC flour (0%, 10%, 20%, 30%, 40%, and 50%) to obtain the following ratios; 100:0, 90:10, 80:20, 70:30, 60:40, and 50:50 that were added equal quantities of sugar, salt, sorghum malt extract, and water. The obtained products were served dry (without added fluid), with water, milk, and warm milk to 15 panelists along with Weetabix Original (commercial control) to evaluate color, consistency, flavor, taste, aftertaste, mouth feel, and overall acceptability using a nine‐point hedonic scale (1 = dislike extremely, 9 = like extremely). The results revealed that the samples were acceptable to the panelists. There were no significant (*P *> 0.05) differences, between the control (Weetabix) and the formulated samples in terms of overall acceptability, when served with water, whereas significant differences (*P* < 0.05) existed when served dry, with milk or warm milk. This new roasting process for producing breakfast cereals offers huge potentials for production of acceptable breakfast cereals enriched with protein and fiber‐rich sources that could be consumed dry, with water, milk, or warm milk.

## Introduction

Breakfast cereals can be categorized into traditional (hot) cereals that require further cooking or heating before consumption and ready‐to‐eat (RTE) (cold) cereals that can be consumed from the box or with the addition of milk Fast (1990); Tribelhorn (1991). Ready‐to‐eat breakfast cereals are increasingly gaining acceptance in most developing countries, and gradually displacing most traditionally established breakfast diets due to convenience, nutritional values, status symbol, improved income and job demands especially among the urban dwellers. According to Jones (2000) instantized and RTE cereals facilitate independence because of their ease of preparation, which means that children and adolescents can be responsible for their own breakfast or snacks. Such foods may need to be reconstituted, preheated in a vessel or allowed to thaw if frozen before consumption, or they may be eaten directly from the box‐pack without further treatment (Okaka 2005). The common cereal products in Nigeria include, NASCO Cornflakes, Good morning flakes, Kellogg's cornflakes, NABISCO flakes, Weetabix, Quaker Oats, Rice crisps, etc. A study has clearly shown that 42% of 10‐year‐olds and 35% of young adults consumed breakfast cereals at nonbreakfast occasions (Haines et al. ), and could be taken dry as snack food, with or without cold or hot milk, based on their location, availability of resources and habits. In recent times, food product developers have incorporated legumes into traditional cereal formulations as nutrient diversification strategy, as well as an effort to reduce the incidence of malnutrition among vulnerable groups. However, this practice is expected to affect both the nutritional and sensory qualities, consequently the acceptability and consumption choice of the end products. Ng'ong'ola‐Manani et al. (2014) determined the sensory properties of fermented pastes of soybeans and soybean–maize blends and successfully established the factors driving consumer liking. In a different study, Atamian et al. (2014) demonstrated through sensory studies that consumer perception of sensory parameters is highly sensitive to sensory attributes. Sensory qualities are known to have clear relationship with product quality, consistency, product development, or consumer acceptance. It can be evaluated from an estimation of total impression the food creates, in the mind of the consumer of the food, applying senses of vision, touch, olfactory, gustatory, and hearing, to evaluate the product's attributes (Das 2005) and generate desired product information that cannot be easily obtained using other methods (Iwe 2002). Oliveira et al. (2013) used sensory evaluation to verify the general acceptance of breakfast cereal with partial replacement of corn grits by the flour of grape seeds and peels from the residues of the wine industry. On the other hand, Kanu et al. 2009 used a 10‐member panel to rate the sensory attributes of breakfast cereal‐based porridge from rice and sorghum, mixed with sesame (*Sesamum indicum L*) and pigeon peas (*Cajanus cajan*) at different percentages. Hersleth et al. (2011) observed that any innovation in a food product affecting consumers' hedonic and/or sensory expectations may also affect its appropriateness, which is a supplement to the hedonic measurement of food acceptance. Thus, this study evaluated the sensory profile of newly formulated instant breakfast cereal products from blends of maize, African yam bean (AYB), and defatted coconut (DC) flour when consumed alone or with/without addition of water, milk, or hot milk.

## Materials and Methods

### Procurement of raw materials

Sound maize grains (*Zea mays L*), AYB seeds (*Sphenostylis stenocarpa*), mature coconut (*Cocos nucifera L*), salt, white sorghum, and sugar were purchased from Ogige market, Nsukka in Enugu State, Nigeria.

### Raw materials preparation

All the grain raw materials were cleaned by removing extraneous materials and sorted to remove shriveled or damaged seeds prior to subjecting them to specific process treatment(s).

### Production of maize, AYB, and DC flour

Maize flour was processed using a modification in the method described by Ihekoronye and Ngoddy (1985) and Okaka (2005). Five kilograms (5 kg) of maize was cleaned and sorted manually, prior to milling into flour using an attrition mill, whereas 5 kg of cleaned/sorted AYB seeds were weighed out using a top loading digital balance (JT302N England). They were washed thoroughly with clean tap water, soaked for 12 h, and boiled for 30 minutes. The beans were dried in a hot air oven (Shellab model VWR‐1370G) at 60°C and milled using attrition mill. The flours obtained were sieved using 0.5‐mm mesh sieve and packaged in polyethylene bags (Enwere ). Three kilograms (3 kg) of freshly dehusked coconut were properly cleaned and cracked to expel the coconut juice/water. The coconut flesh was removed from the shell with the aid of a sharp‐pointed kitchen knife. The brown color of the skin of each coconut piece was scraped off with a knife, whereas the obtained white coconut flesh was grated using a manual grater, homogenized in boiling water (1:4) and filtered through a clean muslin cloth to obtain DC paste that was further dewatered by manual squeezing. Hot water (>70°C) was used to rinse the coconut residue and squeezed to further decrease moisture and fat contents. The DC paste was then dried in the hot air oven (60°C), milled as expressed above and packaged (Sanful 2009).

### Production of sorghum malt

Four kilograms (4 kg) of sorghum grains were steeped in tap water (1:3) for 18 h and germinated on wet jute bag floor for 3 days at room temperature (28 ± 2°C). The green malt was then kilned at 55°C for 8 h and further at 65°C for 16 h until the rootlets was friable, in an oven. The rootlets were separated from the grains. Three‐step decoction method of Okafor and Aniche () was used to mash the sorghum malt during which 70% of the mash was maintained at 55°C for 30 min and at 65°C for 1 h and lastly at 70°C for 1 h. This was strained through a clean muslin cloth and the filtrate (malt extract) stored for use.

### Products formulation

A composite of maize and AYB flour was produced at a ratio of 40:60 and varied with incorporation of graded levels of DC flour (100:0, 90:10, 80:20, 70:30, 60:40, 50:50) (Table 1). The formulations were mixed with equal quantities of sugar, salt, sorghum malt extract, and water to obtain six samples. The samples were roasted at 280°C using a nonstick surface over a heat source.

**Table 1:** Product formulation of breakfast cereals from blends of AYB + maize: defatted coconut flour per 100 g

Ingredient (%)	Sample formulations					
	100:0	90:10	80:20	80:20	60:40	50:50
M + AYB composite flour	84	74	64	54	44	34
Defatted coconut (DC)	–	10	20	30	40	50
Malt extract	10	10	10	10	10	10
Sugar	5	5	5	5	5	5
Salt	1	1	1	1	1	1
Total	100	100	100	100	100	100

AYB, African yam bean; M, maize; DC, defatted coconut flour.

### Sensory evaluation

Sensory evaluation which measures and quantifies the relationship between the sensory characteristics of food and its consumer preferences (Zhang et al. 2011), were used as described below to evaluate the samples (Ihekoronye and Ngoddy 1985).

The six formulated samples were served at 10.00 am to 15 semitrained panelists consisting of students from the Department of Food Science and Technology, University of Nigeria, Nsukka, who are familiar with the sensory attributes of breakfast cereals along with Weetabix original that served as commercial control. Weetabix original was chosen as control because it was available, contained wholegrain and malt extracts amongst others. They were assessed for color, consistency, flavor, taste, aftertaste, mouth feel, and overall acceptability, using a nine‐point hedonic scale questionnaire (1 = dislike extremely, 9 = like extremely). The samples were served alone or with addition of 2.5 parts of milk/water as follows; dry, with water (*t* = 28°C), milk (*t* = 28°C), and warm milk (*t* = 55°C). Portable water was provided for rinsing mouth after tasting each sample, to minimize error and avoid masking perception of the attributes.

### Statistical analysis

The data generated were analyzed with SPSS version 17.0 for Windows (SPSS Inc, Chicago, IL) using One‐Way ANOVA, whereas the means were separated performed using Duncan's multiple range test at *P* = 0.05.

## Results and discussion

According to Okafor and Usman (2014) the proximate composition and energy values of the breakfast cereals ranges (%) as follows: protein (15.68–18.26), moisture (3.38–4.20), fat (1.84–2.02), crude fiber (6.70–9.08), carbohydrates (59.99–62.31), ash (5.29–7.36), and energy (326.63–339.47). The proximate composition may help to influence the perception of the attributes, manifested in the assessors scores.

### Attributes perception of the samples served dry

The result of serving the obtained samples as they were to assessors is presented in Table 2. It shows that there were no significant (*P* > 0.05) differences between the samples in all the attributes evaluated, except the commercial control (G) that was found to be significantly different (*P* <0.05) in terms of color, flavor, taste, consistency, and overall acceptability. In terms of consistency, the sample with 70:30 (D) formulation ranked next to the control sample (Weetabix), whereas 90:10 (B) and 50:50 (F) samples showed closest similarities to the control sample in terms of flavor. The reason for this may be attributed to the strong AYB and coconut flavors which were observed to be outstanding in these samples. In terms of taste, 70:30 sample ranked next to the control sample, although, it showed not significantly (*P* > 0.05) different from other samples. In terms of aftertaste, the judges preferred 90:10 and 50:50 formulations along with the control. This also may be due to the strong taste and flavor of the AYB and DC prominent in these samples, that lingered in the mouth after swallowing. It is also an indication that the processing technique employed in the production of the samples was able to significantly (*P* <0.05) reduce the beany flavor inherent in AYB, thus making the products desirable.

**Table 2:** Sensory scores of breakfast cereals consumed dry (without milk or water)

Sample ratio	Color	Consistency	Flavor	Taste	Aftertaste	Mouth feel	Overall acceptability
100:0	5.73 ± 1.79^a^	6.00 ± 1.36^b^	5.27 ± 1.27^b^	5.60 ± 1.76^b^	5.00 ± 1.65^b^	5.60 ± 1.24^b^	5.93 ± 1.16^b^
90:10	6.73 ± 0.79^a^	6.07 ± 0.59^b^	6.00 ± 1.00^b^	5.87 ± 0.99^b^	5.93 ± 1.22^ab^	6.00 ± 1.07^ab^	5.87 ± 1.25^b^
80:20	6.13 ± 1.13^a^	5.93 ± 0.88^b^	5.60 ± 1.12^b^	5.67 ± 1.04^b^	5.47 ± 1.19^b^	5.80 ± 0.94^ab^	5.67 ± 1.23^b^
70:30	6.53 ± 1.30^a^	6.07 ± 1.33^b^	5.67 ± 1.17^b^	6.07 ± 1.28^b^	5.53 ± 1.25^b^	5.80 ± 1.01^ab^	6.13 ± 1.36^b^
60:40	6.13 ± 1.01^a^	5.80 ± 1.42^b^	5.40 ± 1.35^b^	5.33 ± 1.49^b^	4.93 ± 1.33^b^	5.40 ± 1.35^b^	5.47 ± 1.36^b^
50:50	6.27 ± 1.49^a^	5.53 ± 1.81^b^	5.93 ± 1.16^ab^	5.87 ± 1.12^b^	6.00 ± 1.13a^b^	5.53 ± 1.13^ab^	6.00 ± 0.85^b^
Control	5.67 ± 2.06^a^	7.20 ± 0.94^a^	6.87 ± 1.41^a^	7.07 ± 1.22^a^	6.67 ± 1.50^a^	6.53 ± 1.55^a^	7.13 ± 1.19^a^

Values represent mean ± SD (*n* = 15). Means with different superscripts (a,b,c…) along the same column are significantly (*P* < 0.05) different. Control = Weetabix breakfast cereal. Sample ratio: AYB + maize: defatted coconut fiber.

In terms of mouth feel, samples 100:0 and 60:40 were found to be significantly (*P* < 0.05) different from the control and other samples. In terms of overall acceptability, the evaluation revealed that none of the samples was rejected by the assessors. However, the commercial control was found to be the most acceptable, probably because the assessors were accustomed to the product followed by samples 70:30, 50:50, 100:0, 90:10, 80:20, and finally sample 60:40.

### Attribute perceptions of the samples served with water (*T* = 28°C)

The sensory scores of the samples consumed by placing them in a bowl of water at room temperature (*T* = 28 ± 2°C) is shown in Table 3. Addition of water altered the assessors perception of the samples attributes. The samples and the control were not significantly (*P* >0.05) different from each other in terms of flavor, taste, aftertaste, mouth feel, and overall acceptability. This may be attributed to dissolution of the samples, and neutralization of some of the attributes by the water used to serve the samples. In terms of color, samples 70:30, 60:40, and 50:50 were most preferable. Their scores were significantly (*P* < 0.05) higher than other samples, including the control. In terms of consistency, all the samples except 100:0 formulation were not significantly (*P* > 0.05) different from the control. Consuming the samples with water reduced the differences in the ratings between them and the control. The fact that the samples had closer attributes, suggests that they have the potential of being accepted when introduced to consumers.

**Table 3:** Sensory scores of breakfast cereals served in cold water (28°C)

Sample ratio	Color	Consistency	Flavor	Taste	Aftertaste	Mouth feel	Overall acceptability
100:0	5.25 + 2.08^ab^	5.13 + 2.42^b^	6.79 + 2.12^a^	6.06 + 2.17^ab^	5.12 + 2.21^a^	5.25 + 2.49^ab^	5.44 + 2.42^a^
90:10	5.80 + 2.21^ab^	6.07 + 1.94^ab^	5.27 + 2.34^a^	5.07 + 2.22^b^	6.33 + 2.09^a^	4.80 + 2.14^ab^	5.60 + 2.26^a^
80:20	5.67 + 2.06^ab^	5.80 + 2.24^ab^	6.00 + 2.07^a^	5.27 + 1.94^ab^	5.20 + 2.08^a^	4.60 + 1.99^b^	5.53 + 2.20^a^
70:30	6.53 + 1.60^a^	5.60 + 2.10^ab^	6.60 + 1.45^a^	6.20 + 1.97^ab^	5.60 + 2.50^a^	6.00 + 1.77^ab^	5.53 + 1.99^a^
60:40	6.47 + 2.03^a^	6.13 + 1.92^ab^	6.13 + 1.92^a^	5.27 + 1.88^ab^	5.67 + 1.80^a^	5.67 + 1.84^ab^	6.00 + 1.51^a^
50:50	5.81 + 2.16^a^	5.57 + 2.79^ab^	6.18 + 2.09^a^	6.86 + 1.87^a^	6.36 + 2.09^a^	6.36 + 1.95^ab^	6.07 + 2.89^a^
Control	4.40 + 1.99^b^	7.13 + 2.26^a^	6.47 + 2.26^a^	6.33 + 2.69^ab^	6.40 + 2.82^a^	6.47 + 2.89^ab^	6.47 + 2.77^a^

Values represent mean ± SD (*n* = 15). Means with different superscripts (a,b,c…) along the same column are significantly (*P* < 0.05) different. Control = Weetabix breakfast cereal. Sample ratio: AYB + maize: defatted coconut fiber.

### Attributes perception of the samples served with milk (*T* = 28°C)

The mean sensory scores of consuming the samples with milk shown in Table 4, revealed significant (*P* < 0.05) differences between the samples and the control, in all the attributes except color, probably due to the masking effect of their color by milk. In terms of consistency, samples 100:0 and 90:10 ranked next to the control. This may be as a result of the low content of DC fiber that visibly improved uniformity of these samples, and enhanced their dissolution into tiny particles, which made them more desirable. The 50:50 formulation had the least score, which may be related to the high fiber in the sample that made it less homogenous. In terms of flavor, significant changes (*P* < 0.05) were observed in all the samples. However, the control shared some similarities with 100:0 and 90:10 formulations. These, however, had some similarities with 80:20 and 70:30 formulations. The 60:40 and 50:50 formulations attracted least scores, which may be due to the high level of DC fiber present in them, thus masking all other ingredients. In terms of taste and aftertaste, significant (*P* < 0.05) differences were observed between the samples and the control, which had the highest score, whereas samples 64:40 and 50:50 were scored least. This again may be due to the higher percentage of the DC fiber present in these samples that may have masked all other ingredients, thereby altering their taste. In terms of mouth feel and overall acceptability, all the samples were preferred next to the control, except 50:50 formulation that was least acceptable, probably due to high fiber content.

**Table 4:** Sensory scores of breakfast cereals served with cold milk (28°C)

Sample ratio	Color	Consistency	Flavor	Taste	Aftertaste	Mouth feel	Overall acceptability
100:0	6.40 + 1.30^a^	6.00 + 1.31^b^	6.40 + 0.99^ab^	6.77 + 1.36^b^	6.07 + 1.22^b^	6.00 + 1.13^b^	6.00 + 1.31^bc^
90:10	6.60 + 0.83^a^	5.93 + 0.96^b^	6.27 + 0.70^ab^	6.20 + 0.94^bc^	5.80 + 1.08^b^	6.00 + 1.13^b^	6.13 + 0.74^b^
80:20	6.13 + 1.19^a^	5.73 + 0.96^bc^	5.53 + 1.13^bc^	5.73 + 1.22^bcd^	5.47 + 1.13^b^	5.40 + 0.99^bc^	5.80 + 0.94^bc^
70:30	6.33 + 0.98^a^	5.80 + 1.21^bc^	5.80 + 1.42^bc^	5.60 + 1.55^bcd^	5.40 + 1.35^b^	5.20 + 1.37^bc^	5.53 + 1.46^bc^
60:40	6.20 + 1.01^a^	5.47 + 0.99^bc^	5.13 + 1.06^c^	5.20 + 1.15^d^	5.13 + 1.30^b^	5.07 + 1.22^bc^	5.13 + 1.25^b^
50:50	5.87 + 1.13^a^	5.07 + 0.96^c^	5.33 + 1.45^c^	5.27 + 1.09^cd^	5.20 + 1.26^b^	4.67 + 1.23^c^	5.13 + 0.92^c^
Control	5.93 + 1.98^a^	7.40 + 0.83^a^	7.00 + 1.07^a^	7.33 + 0.89^a^	7.07 + 1.09^a^	7.33 + 0.98^a^	7.47 + 0.83^a^

Values represent mean ± SD (*n* = 15). Means with different superscripts (a,b,c…) along the same column are significantly (*P* < 0.05) different. Control = Weetabix breakfast cereal. Sample ratio: AYB + maize: defatted coconut fiber.

### Attributes perception of the samples served with warm milk (*T* = 55°C)

The mean sensory scores presented in Table 5, shows the influence of serving the samples with warm milk (*T* = 55°C), which was found to alter the attributes perception of the samples compared to the samples served with milk at room temperature (*T* = 28°C). The commercial control sample was the most preferred in all the attributes except color. However, the color of 90:10 formulation was most preferred and significantly (*P* < 0.05) differed from the control sample, which was least preferred, probably because of its darker color compared to the formulated samples. In terms of consistency, all the formulated samples, however, had no significant (*P* > 0.05) differences between them. In terms of flavor, 100:0 and 90:10 formulations were not significantly (*P* > 0.05) different from the commercial control. These two samples shared similarities with 70:30 formulation and with other samples. Laing and Willcox (1983) demonstrated that in binary mixtures, the odor profiles were generally similar to or predictable from the profiles of the components, although any intensity mismatch tended to favor the dominant component at the expense of the weaker item. In terms of taste, samples 100:0, 90:10, 70:30 were preferred alongside the control which were significantly (*P* < 0.05) different from other formulated samples that shared similar characteristics. In terms of aftertaste samples 100:0, 90:10, and 70:30 had no significant (*P* > 0.05) differences with the control. Samples 80:20 and 60:40 were scored below average but shared similarities with samples 100:0 and 50:50, respectively. In terms of mouth feel, only sample 90:10 shared some similarities with the control. This sample also shared some similarities with samples 100:0 and 70:30, but was significantly (*P* < 0.05) different from samples 80:20, 60:40, and 50:50. All the samples except, 60:40 and 50:50 formulations scored above average. In terms of overall acceptability, sample 90:10 shared some similarities with the control as well as with other samples except 60:40 formulation.

**Table 5:** Sensory scores of breakfast cereals served with warm milk (50°C)

Sample ratio	Color	Consistency	Flavor	Taste	Aftertaste	Mouth feel	Overall acceptability
100:0	6.53 + 1.36^ab^	5.67 + 1.40^b^	5.73 + 1.51^bc^	5.47 + 1.36^abc^	5.40 + 1.45^ab^	5.33 + 1.35^bc^	5.80 + 1.36^bc^
90:10	6.80 + 0.68^a^	6.13 + 0.92^b^	6.20 + 0.86^ab^	6.33 + 0.89^ab^	5.87 + 0.92^ab^	6.13 + 0.83^ab^	6.20 + 0.77^ab^
80:20	6.00 + 1.07^ab^	5.53 + 1.25^b^	5.00 + 1.25^c^	5.33 + 1.45^bc^	4.73 + 1.16^c^	5.00 + 1.25^c^	5.47 + 1.36^bc^
70:30	6.53 + 0.83^ab^	5.67 + 1.40^b^	5.53 + 1.51^bc^	5.47 + 1.36^abc^	5.40 + 1.45^ab^	5.33 + 1.35^bc^	5.47 + 1.36^bc^
60:40	6.33 + 0.97^ab^	5.27 + 0.80^b^	4.73 + 1.33^c^	4.73 + 1.16^c^	4.40 + 1.30^c^	4.67 + 0.98^c^	5.07 + 1.10^c^
50:50	6.27 + 1.28^ab^	5.27 + 1.39^b^	5.73 + 1.36^abc^	5.27 + 1.62^bc^	5.00 + 1.65^bc^	4.87 + 1.46^c^	5.33 + 1.20^bc^
Control	5.73 + 1.94^b^	7.40 + 1.01^a^	6.67 + 1.45^a^	6.53 + 1.68^a^	6.40 + 1.76^a^	6.87 + 1.88^a^	6.87 + 1.81^a^

Values represent mean ± SD (*n* = 15). Means with different superscripts (a,b,c…) along the same column are significantly (*P* < 0.05) different. Control = Weetabix breakfast cereal. Sample ratio: AYB + maize: defatted coconut fiber.

## Effect of consumption option on sensory attributes of the samples

**Figure 1:**
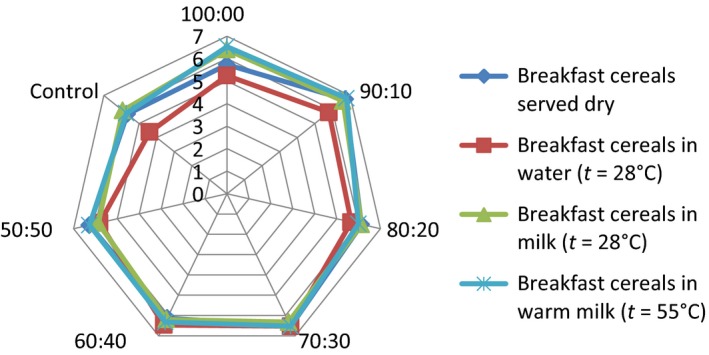
Appearance rating of breakfast cereals served dry, with water (*t* = 28°C), milk (*t* = 28°C), or warm milk (*t* = 55°C).

**Figure 2:**
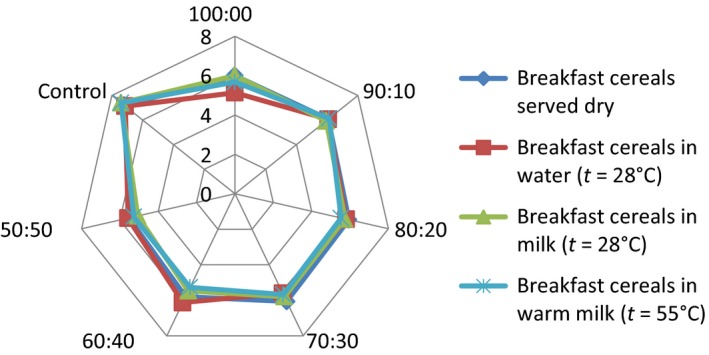
Consistency rating of breakfast cereals served dry, with water (*t* = 28°C), milk (*t* = 28°C), or warm milk (*t* = 55°C).

**Figure 3:**
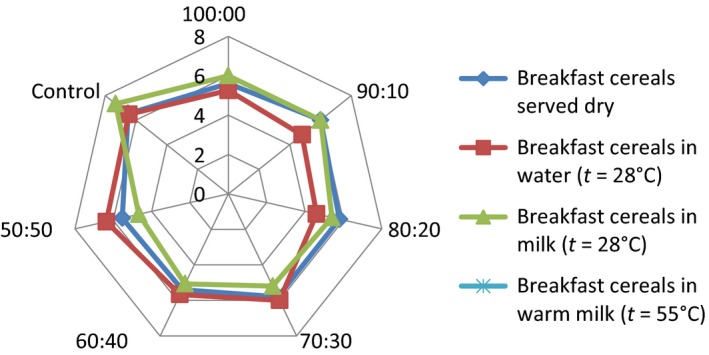
Mouth feel rating of breakfast cereals served dry, with water (*t* = 28°C), milk (*t* = 28°C), or warm milk (*t* = 55°C).

**Figure 4:**
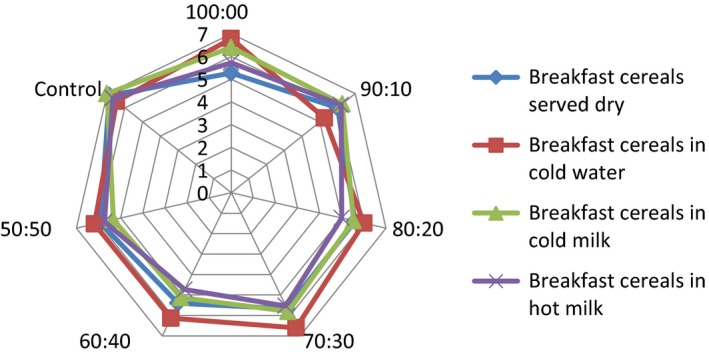
Flavor rating of breakfast cereals served dry, with water (*t* = 28°C), milk (*t* = 28°C), or warm milk (*t* = 55°C).

**Figure 5:**
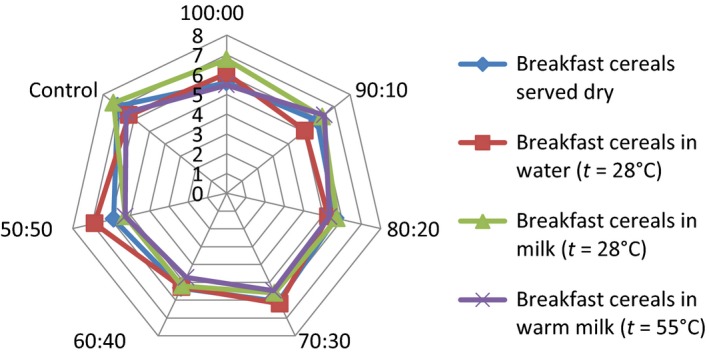
Taste rating of breakfast cereals served dry, with water (*t* = 28°C), milk (*t* = 28°C), or warm milk (*t* = 55°C).

**Figure 6:**
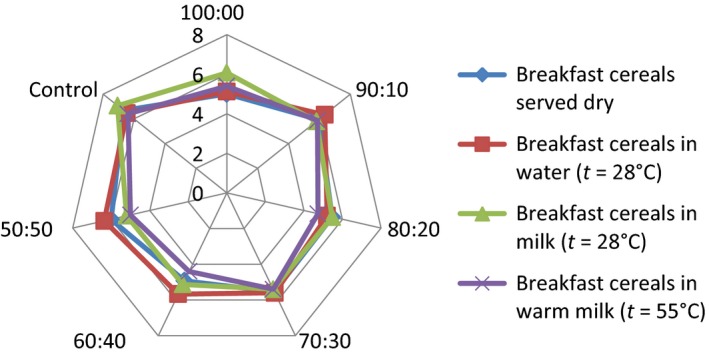
Aftertaste rating of breakfast cereals served dry, with water (*t* = 28°C), milk (*t* = 28°C), or warm milk (*t* = 55°C).

**Figure 7:**
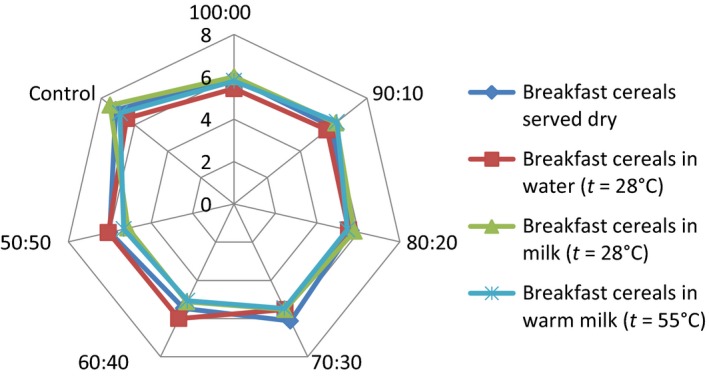
Overall acceptability of breakfast cereals served dry, with water (*t* = 28°C), milk (*t* = 28°C), or warm milk (*t* = 55°C).

The serving of the formulated samples with water, milk, warm milk, or water, influenced the general perception and the ratings of the 15 panelists used for the sensory evaluation. Pictorial representations of the effect of serving option on each of the attributes are shown in Figures 1, 2, 3, 4, 5, 6, 7. The charts revealed that the samples were most preferable when served with water and milk in almost all the attributes, except appearance (Fig. 1) that was rated highest, when served with warm milk. This may be due to complete homogenization of the sample by warm milk, thereby presenting a more uniform appearance. According to Yeu et al. (2008) addition of milk improved the aroma and texture of extruded soy‐based high protein breakfast cereal. The judges, however, awarded low scores to flavor, taste, and aftertaste when served dry, which may be due to the delay in release of flavor and taste of dry samples in the mouth. In terms of overall acceptability, the control sample was most preferable in water followed by eating in dry form, in warm milk and lastly in water. Samples 60:40 and 50:50 were most acceptable when served in water. The warm served samples gelatinized and became very thick, which influenced their lower ratings, relative to the attributes. It is important to note, however, that almost all the perception ratings were above average, for all the serving options applied. Since only human sensory data provide the best model on how consumers are likely to perceive and react to food products in real life (Lawless and Heymann 2010), these findings would help to predict the products consumption pattern when commercialized.

Addition of milk improved aroma and texture acceptance scores and addition of cinnamon flavor improved overall, aroma, and taste acceptance scores. The effect of serving style on consistency and mouth feel (Figs. 2 and 3) revealed that the control was most preferred when served with both cold and hot milk. This may be linked to familiarity of judges with the control sample served with milk, as it is a commercially available product.

## Conclusions

Acceptable RTE breakfast cereals could be produced from blends of maize, AYB, and DC flour, which is evident from the above average scores in almost all the attributes evaluated. In terms of color, they compared favorably and were even more preferred than the commercial control. Most panelists preferred consuming the samples served with either milk at room temperature (*t* = 28°C) or warm milk (*t* = 55°C).

## Conflict of interest

None declared.
